# Pharmacological Inhibitors of the NLRP3 Inflammasome

**DOI:** 10.3389/fimmu.2019.02538

**Published:** 2019-10-25

**Authors:** Ayesha Zahid, Bofeng Li, Arnaud John Kombe Kombe, Tengchuan Jin, Jinhui Tao

**Affiliations:** ^1^Department of Rheumatology and Immunology, The First Affiliated Hospital of USTC, Division of Life Sciences and Medicine, University of Science and Technology of China, Hefei, China; ^2^Division of Molecular Medicine, Hefei National Laboratory for Physical Sciences at Microscale, CAS Key Laboratory of Innate Immunity and Chronic Disease, Division of Life Sciences and Medicine, University of Science and Technology of China, Hefei, China; ^3^CAS Center for Excellence in Molecular Cell Science, Shanghai, China

**Keywords:** NLRP3 inflammasome, inhibitors, MCC950, drug screening, IL-1β

## Abstract

Inflammasomes play a crucial role in innate immunity by serving as signaling platforms which deal with a plethora of pathogenic products and cellular products associated with stress and damage. By far, the best studied and most characterized inflammasome is NLRP3 inflammasome, which consists of NLRP3 (nucleotide-binding domain leucine-rich repeat (NLR) and pyrin domain containing receptor 3), ASC (apoptosis-associated speck-like protein containing a caspase recruitment domain), and procaspase-1. Activation of NLRP3 inflammasome is mediated by highly diverse stimuli. Upon activation, NLRP3 protein recruits the adapter ASC protein, which recruits the procaspase-1 resulting in its cleavage and activation, inducing the maturation, and secretion of inflammatory cytokines and pyroptosis. However, aberrant activation of the NLRP3 inflammasome is implicated in various diseases including diabetes, atherosclerosis, metabolic syndrome, cardiovascular, and neurodegenerative diseases; raising a tremendous clinical interest in exploring the potential inhibitors of NLRP3 inflammasome. Recent investigations have disclosed various inhibitors of the NLRP3 inflammasome pathway which were validated through *in vitro* studies and *in vivo* experiments in animal models of NLRP3-associated disorders. Some of these inhibitors directly target the NLRP3 protein whereas some are aimed at other components and products of the inflammasome. Direct targeting of NLRP3 protein can be a better choice because it can prevent off target immunosuppressive effects, thus restrain tissue destruction. This paper will review the various pharmacological inhibitors of the NLRP3 inflammasome and will also discuss their mechanism of action.

## Introduction

In mammals, the immune system relies on innate immunity and adaptive immunity to protect the host from any external or internal danger (1). The innate immune response utilizes pattern-recognition receptors (PRRs) to sense endogenous or exogenous pathogens (2). A newly identified PRR, which was reported in detail for the first time in 2002, is the inflammasome. It is a high molecular weight protein complex which elicits the activation of inflammatory caspases and processing of pro-interleukin-1β (pro-IL-1β). Inflammasomes are of vital importance in innate immunity because they serve as signaling platforms which are capable of dealing with a plethora of pathogenic products and cellular products associated with stress and damage (3, 4). At present, there are five inflammasomes which are clearly identified, including nucleotide-binding domain leucine-rich repeat (NLR) and pyrin domain containing receptor 1 (NLRP1), NLRP3, and NLR and caspase recruitment domain containing receptor 4 (NLRC4) and the AIM2-like receptors (ALR) family including absent in melanoma 2 (AIM2) (5, 6). This review will describe NLRP3 inflammasome and some reported pharmacological inhibitors targeting this most important inflammasome complex.

## NLRP3 Inflammasome

NLRP3 inflammasome is the best characterized inflammasome at present, named after the NLRP3 protein in the complex which belongs to the NLR family and is also termed as NALP3, CIASI or pyrin domain-containing protein 3 (7). Besides NLRP3 protein, the adapter protein apoptosis-associated speck-like protein containing a caspase recruitment domain (ASC) and procaspase-1 are also part of this inflammasome (8, 9). NLRP3 is a 115 kDa cytosolic protein expressed in monocytes, neutrophils, dendritic cells, lymphocytes, osteoblasts, and epithelial cells (10). It contains three domains which are: a leucine-rich repeat (LRR) at the C-terminal, a central nucleotide-binding and oligomerization domain NACHT which possesses ATPase activity, and a pyrin domain (PYD) at the N-terminal which recruits ASC (11). The danger signal is sensed by the LRR domain which leads to the oligomerization of NLRP3 monomers through their NACHT domains. This is followed by the interaction between the PYD domains of NLRP3 and ASC. Finally, procaspase-1 is recruited into the complex through its CARD domain by ASC which acts as an adaptor protein (12). Recently, Sharif et al. determined the structure of recombinant complex of maltose binding protein (MBP)-tagged NLRP3 protein without pyrin domain and mitotic Ser/Thr kinase NEK7. The cryo EM map showed an earring shape structure composed of curved LRR and globular NACHT domains. The C-terminal lobe of NEK7 interacts with multiple NLRP3 domains including the LRR, HD2 (helical domain 2), and NBD (nucleotide-binding domain). This structure suggests the possibility that NEK7 joins adjacent NLRP3 subunits into bipartite interactions to bring about the activation of NLRP3 inflammasome (13).

NLRP3 Inflammasome recognizes a wide range of stimuli which include various protozoans, e.g., *Plasmodium*, ameba, viruses such as adenoviruses, influenza, and Sendai virus, fungi such as *Saccharomyces cerevisiae* and *Candida albicans*, different bacteria such as *Listeria monocytogenes, Escherichia coli*, and *Staphylococcus aureus* (14). NLRP3 Inflammasome can also respond to damage-associated endogenous factors such as drusen (15), uric acid crystals (16), extracellular adenosine triphosphate (ATP) (17), β-amyloid plaques (11), and islet amyloid polypeptide (18).

**Figure 1:**
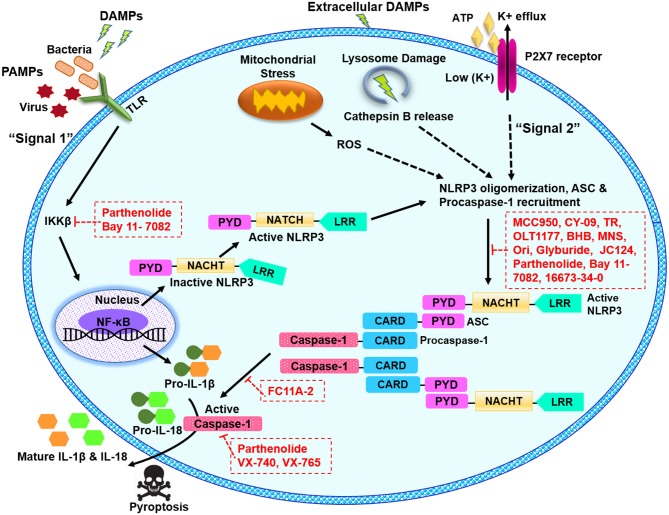
Schematic illustration of NLRP3 inflammasome pathway and potential blockade sites of various pharmacological inhibitors. The signal 1 or the priming signal is mediated by pathogenic PAMPs from bacteria or virus, or sterile DAMPs resulting in NF-κB-dependent upregulation of NLRP3 and pro-IL-1β expression. The signal 2 or activation signal mediated by numerous PAMP or DAMP stimulation, promotes the NLRP3 oligomerization, and recruitment of ASC and pro-caspase-1, leading to the activation of NLRP3 inflammasome complex. NLRP3 can be activated in response to extracellular ATP and K^+^ efflux through the ATP-gated P2X7 channel, in response to cathepsin B release from damaged lysosomes or in response to reactive oxygen species (ROS) released from damaged mitochondria. NLRP3 inflammasome activation results in active caspase-1, which cleaves the proforms of IL-1β and IL-18 into their mature forms. ASC, apoptosis-associated speck-like protein containing a C-terminal caspase recruitment domain; ATP, adenosine triphosphate; BHB, β-Hydroxybutyrate; CARD, caspase recruitment domain; DAMPS, danger or damage associated molecular patterns; IL, interleukin; LRR, leucine-rich repeat; MNS, methylenedioxy-β-nitrostyrene; NACHT, central nucleotide-binding and oligomerization; NF-κB, nuclear factor kappa B; Ori, oridonin; P2X7, P2X purinergic receptor 7; PAMPS, pathogen associated molecular patterns; PYD, pyrin domain; ROS, reactive oxygen species; TLR, toll-like receptor; TR, tranilast.

Activation of NLRP3 inflammasome signaling pathway needs two independent yet parallel steps i.e., priming and activation (19–21). Basal expression of NLRP3 protein and the precursor pro-form of IL-1β is very low, therefore a priming step or “signal 1” initiates the transcription of these targets. Priming step is induced by toll-like receptors (TLRs), myeloid differentiation primary response 88 (MyD88) and/or cytokine receptors, e.g., TNF receptor, which recognize PAMPs or DAMPs and activate the transcription of NLRP3 and pro-IL-1β (14, 22, 23) as illustrated in Figure 1. Recently, many studies have provided strong evidences that priming step is not limited to transcriptional upregulation, post-translational modifications (PTMs) such as ubiquitination and phosphorylation of NLRP3 protein also play critical roles in NLRP3 inflammasome activation (24–26). The second activation step occurs as the primed cell recognizes another stimulus (usually a DAMP) (27, 28).

As a result of the second step, caspase-1 is activated and carries out resultant processing and secretion of IL-1β and IL-18 (29). Various molecular mechanisms to explain the activation of NLRP3 inflammasome have been proposed which include mitochondrial reactive oxygen species (ROS) generation (30, 31), pore formation and potassium (K^+^) efflux (32, 33) and lysosomal destabilization and rupture (30, 34).

## NLRP3 Inflammasome Associated Diseases

Anomalous NLRP3 inflammasome activation is linked with the development of many diseases, especially age-associated ailments for example various metabolic syndromes and metabolic disorders including gout (16), atherosclerosis (35), Alzheimer's disease (AD) (11), and type II diabetes (T2D) (36). Enhanced secretion of IL-1β and IL-18 by NLRP3 inflammasome is associated with the progression of atherosclerotic plaque in atherosclerotic patients and animal models (37–39). NLRP3 inflammasome is involved in experimental autoimmune encephalomyelitis (EAE) in animal models and multiple sclerosis (MS) in humans (40, 41). Inappropriate NLRP3 inflammasome activation is also implicated in Crohn's disease, inflammatory bowel disease (IBD), and ulcerative colitis (42–44). NLRP3 inflammasome is also linked with various cancers, such as colon cancer, breast cancer, melanoma, hepatitis C virus-associated hepatocellular carcinoma, and gastrointestinal cancers (45, 46). In addition to NLRP3 activation anomalies, there are also NLRP3 genetic abnormalities collectively termed as cryopyrin-associated periodic syndromes (CAPS). Gain of function mutations in *NLRP3* gene give rise CAPS disorders, resulting in enhanced IL-1β secretion, and other CAPS specific symptoms (47).

## Pharmacological Inhibition of NLRP3 Inflammasome

The association of NLRP3 inflammasome with the plethora of diseases evokes a substantial interest in the scientific community to discover the effective NLRP3 inflammasome inhibitors. By taking advantage of complex signaling cascade of NLRP3 inflammasome, a diverse range of targets can be used for its inhibition. For example, inhibition of NLRP3 inflammasome activation, suppression of upstream signals, blockade of inflammasome assembly, caspase-1 activation inhibition, blockade of pore-forming protein gasdermin D (GSDMD) cleavage, and neutralizing the inflammatory cytokines produced by the NLRP3 inflammasome can be targeted for potential inhibition of NLRP3 inflammasome. Different mechanisms can be opted to achieve these outcomes for example inhibition of NLRP3 inflammasome assembly, inhibition of P2X7 receptor, inhibition of K^+^ efflux, and ROS scavengers can be used (48–51). Furthermore, NLRP3-NLRP3 interactions or NLRP3-ASC interactions can be disrupted. Inhibitors can be directed at the ATP-binding domain of NLRP3 resulting in blockade of its ATPase activity (52, 53). PTMs of NLRP3 protein and other constituents of the NLRP3 inflammasome are reported as an important step to control its assembly. It can be anticipated that future studies may target the biological components which are involved in these PTMs to inhibit NLRP3 inflammasome. In the recent years, several inhibitors of NLRP3 inflammasome pathway have been reported. Here, we describe some recent pharmacological inhibitors of NLRP3 inflammasome pathway, their proposed mode of action and therapeutic potential (Table 1). Figure 1 depicts the proposed sites of action of these inhibitors provided by *in vitro* and *in vivo* experimental data.

**Table 1:** Potential inhibitors of NLRP3 inflammasome and their targets.

**Agent**	**Target(s)**	**Potential mechanism**	**References**
Glyburide	NLRP3 (indirectly)	Inhibits ATP-sensitive K^+^ channels; downstream of P2X7 resulting in inhibition of ASC aggregation	(48, 54)
16673-34-0	NLRP3 (indirectly)	Induces NLRP3 conformational changes secondary to its activation or binding to ASC	(55, 56)
JC124	NLRP3?	Blocks the expression of NLRP3, ASC, caspase-1, pro-IL-1β, TNFα and iNOS	(57)
FC11A-2	NLRP3 (indirectly)	Interferes with proximity induced autocleavage of pro-caspase-1, suppresses IL-1β/18 release	(58)
Parthenolide	NLRP1, NLRP3 inflammasome, Caspase-1, NF-κB, IKKβ kinase activity	Alkylates cysteine residues in caspase-1 and in ATPase domain of NLRP3, inhibits NLRP3 ATPase activity	(59, 60)
VX-740	Caspase-1	Covalent modification of the catalytic cysteine residue in the active site of caspase-1 resulting in caspase-1 blocking and resultant cleavage of pro-IL-1β/18	(61, 62)
VX-765	Caspase-1	Covalent modification of the catalytic cysteine residue in the active site of caspase-1 resulting in caspase-1 blocking and resultant cleavage of pro-IL-1β/18	(61, 63)
Bay 11-7082	NLRP3, IKK, E2/3 enzymes, PTPs	Alkylates the cysteines in the ATPase domain of NLRP3, inhibits NLRP3 ATPase activity	(59, 64–66)
BHB	NLRP3 (Indirectly)	Inhibits K^+^ efflux resulting in reduced oligomerization of ASC and IL-1β/18 release	(49)
MCC950	NLRP3	Blocks the ATPase domain of NLRP3 resulting in inhibition of canonical and non-canonical NLRP3 inflammasome activation	(67, 68)
MNS	NLRP3	Inhibits NLRP3 ATPase activity by cysteine modification, blocks NLRP3 inflammasome activation	(53)
CY-09	NLRP3	Inhibits NLRP3 ATPase activity, blocks NLRP3 inflammasome activation	(69)
Tranilast	NLRP3	Binds to NLRP3 NACHT domain to block NLRP3-NLRP3 and NLRP3-ASC interaction	(52)
OLT1177	NLRP3	Inhibits NLRP3 ATPase activity, blocks NLRP3 inflammasome activation	(70)
Oridonin	NLRP3	Binds to cysteine 279 of NLRP3 to abolish NLRP3-NEK7 interaction, blocks NLRP3 inflammasome activation	(71)

### Indirect Inhibitors

#### Glyburide

Glyburide is a sulfonylurea drug which is widely used in the United States for the treatment of T2D (72). It inhibits ATP-sensitive K^+^ (K_ATP_) channels in pancreatic β cells (73). One study conducted by Lamkanfi et al. showed that glyburide prevents PAMP-, DAMP-, and crystal-induced NLRP3 inflammasome activation in bone marrow-derived macrophages (BMDMs). Its inhibitory potential seems to be specific for NLRP3 inflammasome, since it did not prevent the IL-1β release from activated NLRC4 or NLRP1 pathway (48). When tested in response to stimuli which work independent of the P2X7 receptor but require TLR4 signaling, glyburide effectively prevented the activation of caspase-1 and 1L-1β secretion, suggesting that it works downstream of the P2X7 receptor (48). It did not block the caspase-1 activation in *S. typhimurium*-infected BMDMs which do not require NLRP3 for caspase-1 activation (74), suggesting that it works upstream of NLRP3 (48). Furthermore, glyburide showed inhibitory activity *in vitro* (48, 75) or *in vivo* (76) during NLRP3 inflammasome activation. However, the *in vivo* doses of glyburide to exert its inhibitory affect are quite high, which cause hypoglycemia, therefore its usage is limited to T2D only (21).

#### 16673-34-0

16673-34-0 is an intermediate substrate produced during glyburide synthesis, however, it lacks the cyclohexylurea moiety of glyburide which is involved in insulin release, therefore, it does not affect glucose metabolism. A study carried out by Marchetti et al. in J774A.1 murine macrophages and primary adult rat cardiomyocytes showed that 16673-34-0 inhibits NLRP3 inflammasome formation, but shows no effect on AIM2 or NLRC4 inflammasome. When tested *in vivo*, it showed positive outcome in mouse models of non-reperfused and reperfused acute myocardial infarction. 16673-34-0 was tested with multiple diverse stimuli of NLRP3 inflammasome, independent of which stimuli is used, inhibitory effects of 16673-34-0 remained the same suggesting that it interferes with downstream events involved in either NLRP3 conformational changes secondary to activation or binding to ASC (55, 56). However, the exact mechanism of inhibition is not completely clear and additional studies are needed to fully determine its inhibitory potential.

#### JC124

Kuwar et al. recently developed a novel small molecule JC124, through structural optimization of glyburide. JC124 was rationally designed to remove the potential hypoglycemic effects of glyburide. They explored the potential of JC124 for traumatic brain injury (TBI) therapy and it was demonstrated to exert significant anti-inflammatory effect to protect the injured brain following TBI. JC124 treatment significantly reduced the expression of NLRP3, ASC, caspase-1, pro-IL-1β, TNFα, and inducible nitric oxide synthase (iNOS). This targeting of NLRP3 inflammasome activation and its downstream neuroinflammatory cascade is suggested to confer JC124 its protective effect for TBI (57). It blocked ASC aggregation, caspase-1 activation, and IL-1β secretion. JC124 showed protective effects in a mouse model of acute myocardial infarction (77) and in transgenic AD models (77, 78). Further studies aiming at determining the efficacy of JC124 will render more information for its translational value.

#### FC11A-2

Liu et al. investigated a synthetic small molecular compound, 1-ethyl-5-methyl-2-phenyl-1H-benzo[d]imidazole, which is also known as FC11A-2, for its inhibitory potential of NLRP3 inflammasome. FC11A-2 was examined in THP-1 cells and in mouse model of dextran sulfate sodium (DSS)-induced experimental colitis, and it showed highly effective outcomes by repressing IL-1β/18 release. FC11A-2 hindered the proximity-induced autocleavage of procaspase-1, eventually resulting in reduced amount of activated caspase-1, by a pathway which is independent of activation of NF-κB (58).

### Inhibitors for the Constituents of NLRP3 Inflammasome

#### Parthenolide

Parthenolide is a plant sesquiterpene lactone and has numerous anti-inflammatory properties, therefore, it is utilized in herbal medicines of various inflammatory diseases (79). It inhibited caspase-1 activation in response to NLRP1, NLRC4, and NLRP3 stimulation by alkylating many cysteine residues of caspase-1. Parthenolide can also target ATPase activity of NLRP3 protein directly, probably through cysteine modification (59). However, it had poor solubility and bioavailability, therefore now its water soluble analogs are being evaluated (80, 81).

#### VX-740 and VX-765

VX-740 (Pralnacasan) and its analog VX-765 are peptidomimetic inhibitor of caspase-1. They are prodrugs which are metabolized by plasma esterases to their corresponding aldo-acids (63, 82). Both compound act by covalent modification of the catalytic cysteine residue in the active site of caspase-1, hence they block caspase-1 and resultant cleavage of pro-IL-1β/18 (61). VX-740 showed good results for the treatment of rheumatoid arthritis (RA) and osteoarthritis (OA) in mice models (62). In phase I and II clinical trials in RA patients, it exhibited significant anti-inflammatory effects with good pharmacokinetics profile (83, 84). However, hepatic toxicity in animals after its long-term exposure led to discontinuation of further development (85). VX-765 showed even higher potency for RA and also showed reduction in IL-1β/18 in mouse model of dermatitis. It also had positive outcomes for treatment of epilepsy and psoriasis in mice and was announced to undergo clinical trial (63, 86). Some recent findings have reported that VX-765 helped in alleviating the cognitive impairment and severity of AD in mice (87). It also lowered myocardial infarction and preserved ventricular function in mice (88).

#### Bay 11-7082

Bay 11-7082 is a phenyl vinyl sulfone, it inhibits NF-κB pathway through blockade of kinase activity of IKKβ. It inhibits its target proteins using alkylation of essential nucleophilic residues, for example cysteines. Studies with NG5 cells and mouse primary BMDMs showed that bay 11-7082 prevents the organization of ASC pyroptosome and NLRP3 inflammasome function through alkylation of cysteine residues of NLRP3 ATPase region. Importantly, it showed selective inhibition of NLRP3 inflammasome as compare to other inflammasomes (59). Recently, vinyl sulfone derivatives were used as antiparasitic agents in dogs and mice (89), these preclinical trials revealed that these compounds are well-tolerated, non-mutagenic and have suitable pharmacokinetic profiles. They also permeate cell membrane easily (59). Bay 11-7082 and other vinyl sulfone/sulfonate compounds provides an applicable framework for the future design.

#### β-Hydroxybutyrate (BHB)

ß-hydroxy butyrate (BHB) is a ketone metabolite, which was tested by Youm et al. for NLRP3 inflammasome blockade. It affectively lowered the production of IL-1ß and IL-18 in human monocytes in response to activated NLRP3 inflammasome, without interfering with activated AIM2 or NLRC4 inflammasome. Treatment of BMDMs from mouse models of familial cold auto inflammatory syndrome (FCAS) and Muckle–Wells syndrome (MWS) with BHB dose-dependently inhibited constitutive NLRP3 inflammasome activation. BHB is effective only for canonical activation during which it inhibits K^+^ efflux and reduces the oligomerization and speck formation of ASC. It blocks the activation of NLRP3 inflammasome independent of ROS, AMP-activated protein kinase, glycolytic inhibition, or autophagy (49). From these findings it can be anticipated that pharmacological or dietary attempts to raise BHB level may reduce the severity of NLRP3-mediated chronic inflammatory diseases.

### Direct Inhibitors of NLRP3 Protein

#### MCC950

A diarylsulfonylurea-containing compound termed as MCC950, is considered one of the most potent and selective inhibitor of NLRP3 inflammasome. There is an extensive consideration in the development of MCC950 as a treatment for the NLRP3-driven disorders. MCC950 was previously reported to block the processing of IL-1β by caspase-1 (54), later it was described by Coll et al. that in mouse and human macrophages, MCC950 has the potential to block both canonical and non-canonical NLRP3 inflammasome activation and IL-1β production by abrogating ASC oligomerization. Notably, MCC950 had no effect on AIM2, NLRC4, or NLRP1 inflammasome activation (67, 90, 91). Another latest study has reported that MCC950 directly binds to the NLRP3 NACHT domain's Walker B motif, and blocks the hydrolysis of ATP and formation of NLRP3 inflammasome (68). Very recently, a preprint paper at BioRxiv has reported by utilizing photoaffinity labeling and iBody technology that MCC950 interacts with the NACHT domain of wild type NLRP3. The binding was lessened in most of CAPS-related NLRP3 mutants, moreover, in two mouse models of CAPS, MCC950 did not inhibit the NLRP3-driven inflammatory pathology. This study implies that MCC950 may only be effective in inflammation driven by wild type NLRP3 protein, but not in ailments driven by CAPS-related NLRP3 mutants (92).

MCC950 was reported to lower skin and pulmonary inflammation in mice (93) and some other *in vivo* experiments in mouse model of human MS showed that MCC950 alleviates the severity of EAE (67). Oral treatment of MCC950 rescued the dopaminergic degeneration in a mouse model of Parkinson's disease (PD) (94). Future studies are needed to warrant the exact potential of MCC950.

#### 3,4-Methylenedioxy-β-nitrostyrene (MNS)

A potent NLRP3 inhibitor, 3,4-Methylenedioxy-β-nitrostyrene (MNS) was found through screening a kinase inhibitory library by He et al. By utilizing immunoprecipitation, mass spectrometry, and mutational studies, it was demonstrated that MNS binds to the LRR and NACHT domains and suppresses ATPase activity of NLRP3, while the activation of AIM2 or NLRC4 inflammasomes was unaffected by it. MNS may directly target the cysteine(s) of NLRP3 as implicated by its inhibition of ATPase activity of NLRP3 (53). Future studies on MNS may confer additional insights on this potential inhibitor.

#### CY-09

Jiang et al. identified an effective and direct inhibitor of NLRP3 which showed significant inhibition of NLRP3 inflammasome *in vivo* in mice models and *ex vivo* in human cells (52). CY-09 is an analog of CFTR(inh)-172 (C172), which inhibits the cystic fibrosis transmembrane conductance regulator (CFTR) channel (95). CY-09 lacks CFTR-inhibitory activity (96). In BMDMs primed with LPS, CY-09 dose-dependently blocked the ATP, monosodium urate (MSU), and nigericin-induced activation of caspase-1 and resultant release of IL-1β. Its inhibitory effect is not dependent on signal 1 and NLRP3 post-translational modification (ubiquitination). Mechanistically, it directly interacts with the NLRP3 Walker A motif to eliminate the ATP binding of NLRP3, however, it does not affect NLRP1, NLRC4, RIG-1, or NOD2 (52).

CY-09 demonstrated outstanding preventive or therapeutic properties in the mice models of gout, T2D, and CAPS. Most importantly, it exhibited a promising pharmacokinetic profile and showed good oral bioavailability, safety, and stability. Nonetheless, more studies are required to broaden its full potential (52).

#### Tranilast

Tranilast (N-[3′,4′-dimethoxycinnamoyl]-anthranilic acid, TR) is a tryptophan metabolite analog which showed inhibitory potential for homologous passive cutaneous anaphylaxis (97). TR is a fairly safe compound and its high doses showed appropriate tolerance levels when tested in patients (98, 99). It showed inhibitory effect for NLRP3 inflammasome but not for NLRC4 or AIM2 inflammasome. TR impaired the endogenous NLRP3-ASC interaction but did not affect the NLRP3-NEK7 interaction, raising the possibility that it targets NLRP3 directly. Indeed, it was demonstrated to bind to the NLRP3 NACHT domain and, abolish the direct NLRP3-NLRP3 interaction (52). Moreover, TR does not impede with the upstream signaling events of NLRP3 inflammasome, e.g., expression of NLRP3 and pro-IL-1β, ROS production, K^+^ efflux, chloride efflux, and mitochondrial damage. TR has demonstrated significant therapeutic and preventive outcomes in gout, CAPS, and T2D mice models (69). Considering the high safety of TR in clinic, it can be of significant importance for treating NLRP3-driven diseases.

#### OLT1177

OLT1177 is an active β-sulfonyl nitrile compound, which cleared phase I clinical trial for the treatment of degenerative arthritis successfully, and now being evaluated under phase II clinical trial (100).

A study in mice model of MSU- and zymosan-induced arthritis by Marchetti et al. demonstrated that OLT1177 has the potential to lower the neutrophil infiltration and joint swelling, as well as to inhibit the secretion of IL-1β and IL-6. In *in vitro* studies, OLT1177 blocked both canonical and non-canonical activation of NLRP3 inflammasome and showed direct binding with NLRP3 to block its ATPase activity. Moreover, in monocytes from CAPS patients, it lowered caspase-1 activity and resultant IL-1β secretion, and reduced LPS-induced systemic inflammation in mice. Significantly, OLT1177 did not inhibit NLRC4 or AIM2 inflammasome. OLT1177 was given orally to the healthy subjects in phase 1 trials, and it showed good safety and tolerance levels. Additionally, it had long half-life and did not show any organ or hematological toxicity at various doses (70). Thus, OLT1177 seems to have a significant potential to treat NLRP3-related diseases.

#### Oridonin

Oridonin (Ori) is a bioactive ent-kaurane diterpenoid, a main component of herbal plant *Rabdosia rubescens*, which is extensively utilized in traditional Chinese medicine (101, 102). There are a number of anticancer activities which have been associated with Ori, such as cell cycle arrest, angiogenesis suppression and apoptosis induction (103, 104). It is reported to inhibit the NF-κB or MAPK activation and repress the release of inflammasome-independent proinflammatory cytokines release (105–107). Furthermore, it has good therapeutic effects on neuroinflammation, sepsis and colitis (108–110). He et al. reported that Ori interacts with the cysteine 279 of NLRP3 NACHT domain through a covalent bond, abolishes NLRP3-NEK7 interaction, and inhibits consequent activation of NLRP3 inflammasome. The inhibitory effects of Ori are limited to NLRP3 inflammasome only and it does not inhibit AIM2 or NLRC4 inflammasome activation. When used in mice models of T2D, peritonitis and gouty arthritis, Ori exhibited significant preventive, and therapeutic effects (71). Thus, it can be anticipated that future studies may establish Ori as a clinically applicable inhibitor of NLRP3 inflammasome.

## Concluding Remarks

NLRP3-induced pyroptosis and IL-1β/18 secretion is linked to various diseases. The extent to which NLRP3 inflammasome activation contributes to the pyroptosis is still unclear, however, NLRP3 activation does results in pyroptosis which in turn can cause serious injury to vital organs (111). At present, to treat NLRP3-associated diseases, many drugs are available which block IL-1β such as neutralizing IL-1β antibody canakinumab, recombinant IL-1 receptor antagonist anakinra, and the soluble decoy IL-1 receptor rilonacept. These biological agents are being used to treat CAPS and other diseases associated with IL-1β (112). However, activated NLRP3 inflammasome does not produce only IL-1β, there are other cytokines such as IL-18 which may also contribute to the NLRP3-associated disorders (113, 114). Moreover, IL-1β production can be mediated by other inflammasomes or by inflammasome-independent pathways; thus inhibitors aimed at IL-1β can result in unintentional immunosuppressive effects. Therefore, pharmacological inhibitors which specifically target the NLRP3 inflammasome only could be a better option for treatment of NLRP3-associated diseases. NLRP3-induced pyroptosis has been reported by many recent studies as a critical mechanism contributing to the NLRP3 inflammasome related pathologies (115, 116). Emerging evidences have reported GSDMD as an executive protein responsible for pyroptosis (117, 118), making it an attractive therapeutic target for curing NLRP3-induced pyroptosis associated diseases. Future studies should take advantage of now available structure of NLRP3 and focus on the development of structure-guided direct inhibitors with improved specificity and potency. Furthermore, nanobodies (Nbs) are now being explored extensively as therapeutics due to their high specificity, stability, and low toxicity (119, 120). It can be anticipated that Nbs may also be evaluated for NLRP3 inflammasome inhibition. In the past decade, great leaps forward were made to determine the structure of NLRP3 inflammasome, its activation mechanisms and its contribution to the initiation and progression of different diseases. Moreover, many small molecule inhibitors for NLRP3 inflammasome have been reported and some of them have shown remarkable therapeutic potential. However, none of them is currently approved by food and drug administration (FDA) or other agents. Current research should focus on the development of specific, small-molecular inhibitors of NLRP3 inflammasome which have improved pharmacokinetic properties, can penetrate the blood brain barrier more readily and be more cost-effective.

## Author Contributions

AZ prepared the draft. All authors revised the draft.

### Conflict of Interest

The authors declare that the research was conducted in the absence of any commercial or financial relationships that could be construed as a potential conflict of interest.
